# H3K27-Altered Diffuse Midline Glioma of the Brainstem: From Molecular Mechanisms to Targeted Interventions

**DOI:** 10.3390/cells13131122

**Published:** 2024-06-28

**Authors:** Leo F. Nonnenbroich, Samantha M. Bouchal, Elena Millesi, Julian S. Rechberger, Soumen Khatua, David J. Daniels

**Affiliations:** 1 Department of Neurologic Surgery, Mayo Clinic, Rochester, MN 55905, USA; leo.nonnenbroich@kitz-heidelberg.de (L.F.N.); rechberger.julian@mayo.edu (J.S.R.); 2 Hopp Children’s Cancer Center, Heidelberg (KiTZ), 69120 Heidelberg, Germany; 3 Clinical Cooperation Unit Pediatric Oncology, German Cancer Research Center (DKFZ) and German Consortium for Translational Cancer Research (DKTK), 69120 Heidelberg, Germany; 4 Department of Molecular Pharmacology and Experimental Therapeutics, Mayo Clinic, Rochester, MN 55905, USA; 5 Division of Plastic and Reconstructive Surgery, Department of Surgery, Mayo Clinic, Rochester, MN 55905, USA; 6 Research Laboratory of the Division of Plastic and Reconstructive Surgery, Department of Surgery, Medical University of Vienna, 1090 Vienna, Austria; 7 Department of Pediatric Hematology/Oncology, Section of Neuro-Oncology, Mayo Clinic, Rochester, MN 55905, USA; skhatua.bappa@gmail.com

**Keywords:** diffuse midline glioma, H3K27-altered, H3K27M, DIPG, chemotherapy, targeted therapy, drug delivery, blood–brain barrier, brain tumor, glioma

## Abstract

Pediatric high-grade gliomas are a devastating subset of brain tumors, characterized by their aggressive pathophysiology and limited treatment options. Among them, H3 K27-altered diffuse midline gliomas (DMG) of the brainstem stand out due to their distinct molecular features and dismal prognosis. Recent advances in molecular profiling techniques have unveiled the critical role of H3 K27 alterations, particularly a lysine-to-methionine mutation on position 27 (K27M) of the histone H3 tail, in the pathogenesis of DMG. These mutations result in epigenetic dysregulation, which leads to altered chromatin structure and gene expression patterns in DMG tumor cells, ultimately contributing to the aggressive phenotype of DMG. The exploration of targeted therapeutic avenues for DMG has gained momentum in recent years. Therapies, including epigenetic modifiers, kinase inhibitors, and immunotherapies, are under active investigation; these approaches aim to disrupt aberrant signaling cascades and overcome the various mechanisms of therapeutic resistance in DMG. Challenges, including blood–brain barrier penetration and DMG tumor heterogeneity, require innovative approaches to improve drug delivery and personalized treatment strategies. This review aims to provide a comprehensive overview of the evolving understanding of DMG, focusing on the intricate molecular mechanisms driving tumorigenesis/tumor progression and the current landscape of emerging targeted interventions.

## 1. Introduction

Central nervous system (CNS) tumors are the most frequently diagnosed solid tumors and are the leading cause of disease-related death in children [1,2]. High-grade gliomas of the brainstem represent 10–20% of pediatric CNS malignancies and are characterized by a particularly poor prognosis [3,4]. The vast majority of high-grade brainstem gliomas that were once identified as diffuse intrinsic pontine gliomas (DIPG) are now categorized as H3 K27-altered diffuse midline gliomas (DMG) in the most recent WHO classification of CNS tumors. These universally fatal tumors are characterized by a set of distinct molecular alterations that impact the lysine residue at position 27 (K27) of the histone H3 tail. These modifications ultimately contribute to DMG tumorigenesis and their aggressive clinical behavior [5].

Whereas most other pediatric cancers have seen improvements in survival metrics, the prognosis for DMG patients is still dismal, with reported median overall survival (OS) rates of less than 12–15 months [6,7]. Given the delicate location of DMG in the brainstem and its infiltrative growth patterns, these tumors may be amenable to biopsy but are not suitable for surgical resection [8,9]. Unlike other gliomas, chemotherapeutic treatment approaches have yet to improve patient outcomes in DMG [10]. Radiation therapy represents the current mainstay of DMG therapy, although it is considered palliative and is associated with some morbidity [11]. DMGs are usually diagnosed by their unique clinical course, neurological symptoms, and radiological appearance. However, the dire need for improved understanding of molecular biology and the development/testing of novel therapies have led to the increased use of fine needle biopsies in clinical practice [12,13]. Access to DMG tumor material not only allows for improved patient stratification and histopathologic diagnosis but also provides patient tissue specimens that can be used to study DMG biology [14,15]. Sequencing of DMG patient tissue (biopsy and autopsy specimens) has been vital for the discovery of the now characteristic H3 lysine 27 to methionine (K27M) mutation and defining biologically distinct subgroups of DMG, among other milestones. This review summarizes the molecular alterations thought to drive DMG tumorigenesis as well as the status of recently developed targeted DMG therapies.

## 2. Mechanisms Driving Tumorigenesis of H3 K27-Altered DMG

**Figure 1:**
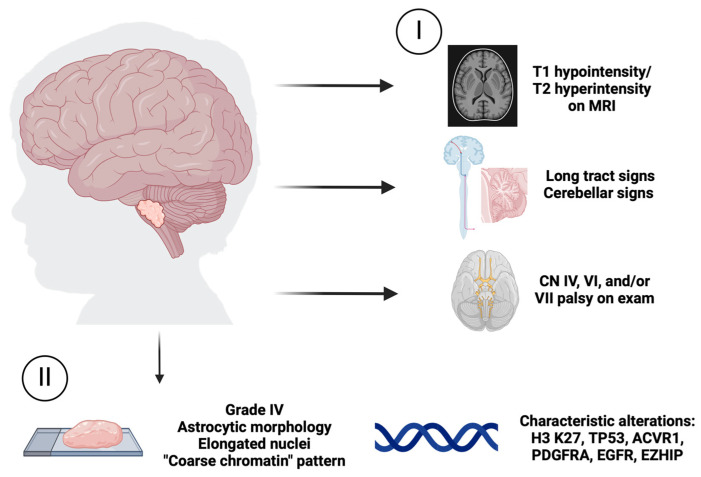
Clinical and pathological features of H3 K27-altered diffuse midline glioma (DMG). I. In the clinic, DMG can be diagnosed using a combination of imaging and physical exam findings. Typical findings include T1 hypointensity/T2 hyperintensity on MRI paired with long tract signs, cerebellar signs, and/or palsies of CN IV, VI, and VII on clinical exam. II. Histopathological features of DMG may include astrocytic morphology, elongated nuclei, a coarse chromatin pattern, and other aggressive features. However, DMG tumors are designated WHO Grade IV, regardless of histological features. Characteristic genetic alterations can be identified in patient tumors.

A diagnosis of DMG may be accomplished using imaging and clinical criteria only (i.e., T1 hypointensity/T2 hyperintensity on magnetic resonance imaging (MRI) paired with long tract signs, cerebellar signs, and/or palsies of CN IV, VI, and VII on clinical exam). However, DMG also exhibits unique histopathologic features, including astrocytic morphology and elongated nuclei with a “coarse chromatin” pattern [16]. H3K27M DMG tumors may exhibit high-grade malignant features but are designated WHO IV irrespective of exact histologic features due to their aggressive clinical behavior [17] (Figure 1).

With growing access to DMG tumor material, more advanced histopathological diagnostic tools, such as DNA-sequencing (DNA-seq) or immunohistochemistry (IHC), can contribute to a more refined diagnosis [13,17]. The 2021 WHO classification of tumors of the CNS defines the following genes/molecular alterations as “characteristically altered” in DMG: H3 K27, TP53, ACVR1, PDGFRA, EGFR, and EZHIP [5]. Landmark studies published in 2012 identified histone H3 mutations as molecular drivers underlying DMG tumorigenesis [18,19]. These findings represented a major breakthrough in our understanding of the pathophysiological underpinnings of this devastating tumor [18,19,20]. Wu et al. found that the H3 K27M mutation leads to a loss of H3 K27 trimethylation (H3 K27me3), although the exact mechanisms underlying this phenomenon remain poorly understood. Whole exome sequencing of primary pediatric GBM samples revealed recurrent mutations in histone 3 variant H3.3 (H3F3A) and subsequent amino acid substitutions at K27 with methionine (K27M), a substitution with arginine at G34 (G34R), or with valine (G34V) [18]. Mutations occurring in HIST1H3B (encoding H3.1) have also been identified in DMG [18].

Together, the H3 K27M mutation induces global epigenetic changes, which ultimately lead to altered gene expression programs that “prime” cells for tumorigenesis [21]. A 2022 study by Kfoury-Beaumont et al. suggests that H3 K27M directly interferes with differentiation and promotes clonogenicity in human embryonic stem cells [22]. Current studies suggest the binding and association of H3 K27M to EZH2 cause separation and non-functional recruitment of PRC2, a mediator of H3 K27 methylation [23]. As a result, histone acetylation is increased, while H3 K27 methylation is globally suppressed [24]. Additional alterations in platelet-derived growth factor receptor alpha (PDGFRA), found in 30% of DMGs, and tumor protein 53 (TP53), described in 42% of DMG tumors, are associated with DMG tumorigenesis [6,25,26]. Specific molecular alterations may be associated with DMG tumor anatomical location as well as survival outcome [27]. In a recent study, Roberts et al. demonstrated that enrichments in mitogen activated pathway kinase (MAPK) alterations were associated with extended survival in DMG patients compared to DMG patients without MAPK alterations [28]. In line with these findings, the 2021 WHO classification of tumors according to the CNS criteria divides DMG into three subtypes: DMG with H3 K27M mutation, DMG with H3 K27M-wildtype with overexpression of EZHIP protein overexpression, EGFR mutation, or other molecular changes. Also, a characteristic methylation profile in accordance with one of the described DMG subtypes may be used as a diagnostic criterion [5,29].

Efforts to characterize DMG precursor cells identified neural precursor cell populations located in the ventral pons that may be temporally and spatially linked to DMG occurrence. Interestingly, these precursor cells showed upregulated hedgehog (Hh) pathway activity. Additionally, a significant portion of this cell population was Olig2+, which is commonly cited as a marker of oligodendrocyte precursor cells [30,31].

## 3. Treatment Strategies in H3 K27-Altered DMG: Translational Advances and Challenges

**Figure 2:**
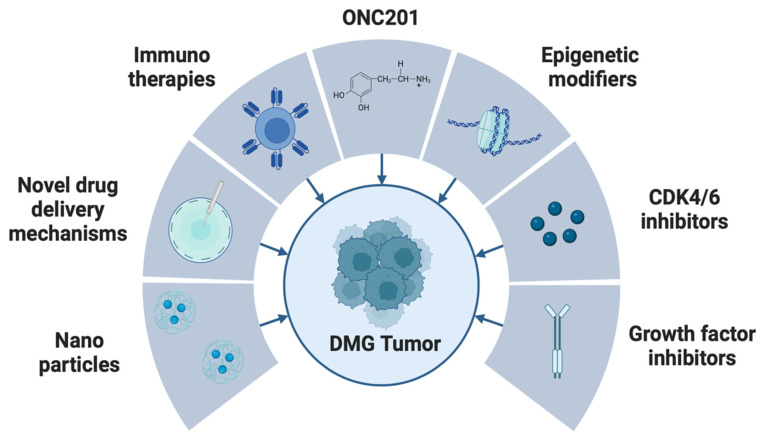
Therapeutic options that are being actively explored in DMG.

Novel, more effective therapeutic strategies are urgently needed to improve patient prognosis for patients with H3K27M-altered DMG. Broad chemotherapeutic strategies have yet to demonstrate benefit in clinical trials for DMG. As such, targeted therapies specifically addressing DMG molecular pathology may be of more value [9,11]. Targeted therapies currently under preclinical investigation in DMG include small-molecule inhibitors targeting mutated histone modifiers, epigenetic modifiers, and receptor tyrosine kinases, among others. Immunotherapeutic strategies such as immune checkpoint inhibitors and chimeric antigen receptor (CAR) T-cells are also being explored [32]. In addition to the development of novel therapeutic targets, drug delivery to DMG tumors remains a major challenge. The blood–brain barrier (BBB) is well preserved in DMG compared to other brain tumors and poses a significant obstacle in drug delivery; as a result, drugs often fail to reach therapeutic doses intratumorally [33]. Various techniques that bypass or disrupt the BBB (such as convection enhanced delivery (CED) and focused ultrasound (FUS)) have shown promising results and may be capable of overcoming this substantial barrier in drug delivery [34,35,36] (Figure 2).

## 4. Beyond Chemotherapy—Targeted Therapy in H3 K27-Altered DMG

### 4.1. Pathway Inhibitors

Vascular endothelial growth factor (VEGF) and other growth factors have been shown to play a role in the pathogenesis of gliomas, prompting interest in the use of growth factor inhibitors as therapeutics. Growth factor inhibitors have also been investigated in DMG. However, clinical investigations of vandetanib, a VEGFR2 and epidermal growth factor receptor (EGFR) inhibitor, have not shown meaningful survival benefits. DMG patients receiving vandetanib did not experience survival benefits even with radiation therapy [37]. Efforts to evaluate the monoclonal VEGFR antibody bevacizumab in DMG have also failed to achieve consistent, beneficial results [38,39,40,41]. A combination of bevacizumab and cetuximab (a monoclonal IgG1 EGFR antibody) administered as a super-selective intraarterial cerebral infusion (SIACI) was tolerated well. The efficacy of this drug combination and BBB disruption via SIACI is an active area of investigation [42].

Preclinical investigations of dasatinib, a platelet-derived growth factor receptor A (PDGFRA) inhibitor, showed promising activity in preclinical DMG models; furthermore, its in vitro anti-tumorigenic effect was enhanced by co-administration of the c-Met inhibitor cabozantinib [43]. However, no survival or clinical benefit was found for dasatinib, nor was there an observed benefit for the combination of dasatinib and cabozantinib. These treatments were also not tolerated well [44,45]. A study investigating the topoisomerase I inhibitor irinotecan and the EGFR inhibitor erlotinib in combination with bevacizumab found minimal beneficial effects of this combination in high-risk DMG patients [41].

As some DMGs harbor amplifications in cyclin-dependent kinase (CDK) 4 and 6 genes, the CDK4/6-inhibitor ribociclib has been investigated in DMG tumors with this alteration [46]. Furthermore, a novel CDK4/6 inhibitor (YF-PRJ8-1011) synthesized by Zuo et al. exhibits superior efficacy both in vitro and in vivo to palbociclib, penetrates the blood–brain barrier relatively well, and synergizes with radiotherapy in DMG xenograft models [47]. A Phase 1/2 study (NCT02607124) with ribociclib, following radiotherapy in DMG, demonstrated feasibility and a 1-year OS of 89% of patients [48]. A Phase I trial to determine the pharmacokinetics of abemaciclib in DMG via intratumoral microdialysis (NCT05413304) is currently underway [49].

In light of the altered epigenome of DMG, epigenetic modifiers such as histone deacetylase complex inhibitors (HDACi) have been identified as potent inhibitors of DMG cell line growth in large drug screens [50]. Several studies confirmed the efficacy of the HDACi panobinostat in murine DMG models, although the reported beneficial effect on survival was inconsistent between studies [51,52,53]. Recently, the combination of panobinostat with the proteasome inhibitor marizomib led to improved survival in murine DMG models [54]. Clinical trials to investigate the potential uses of panobinostat, HDACi, and vorinostat are underway [55,56]. Inhibitors of the EZH2 protein are also being investigated in DMG. Specifically, the EZH2 inhibitor GSK126 decreases DMG cell proliferation in vitro and may be combined with statins to inhibit tumor growth in in vivo models of DMG [57]. However, caution with these agents is warranted, as EZH2 was recently demonstrated to play a tumor-suppressive role in DMG pathogenesis [58]. Finally, an expanded epigenetic inhibitor library screen performed by Brown et al. identified protein arginine methyltransferase (PRMT) inhibitors as potential therapeutic targets in DMG [59]. LLY-283, an inhibitor of PRMT5, was shown to decrease the invasion and viability of DMG in vitro but failed to improve survival in vivo. Future work is needed to determine whether PRMT inhibitors can be used in combinatorial approaches to augment existing therapies.

### 4.2. ONC201

The dopamine receptor D2 inhibitor (DRD2i) and caseinolytic protease P (ClpP) agonist ONC201 can induce mitochondrial damage within tumor cells; this compound has been shown to generate reactive oxygen species and induce apoptosis in DMG cell lines and in vivo models. Interestingly, ONC201 also induced DMG cell line maturation and differentiation [60]. The clinical potential of ONC201 for the treatment of DMG was first demonstrated by a case report from Hall et al., in which a patient receiving ONC201 therapy experienced stable disease at 18 months as well as clinical improvement [61]. These encouraging findings and the success of early-phase clinical trials led to an expanded phase II trial of ONC201 (NCT02525692), which resulted in a median OS of 16 months [62]. Additionally, German-sourced ONC201 (gsONC201) showed promising in vitro and in vivo activity. A clinical study of the compound in 28 H3K27M DMG patients found a median OS of 18 months; patients who underwent re-irradiation in addition to the GsONC201 had a median OS of 22 months, whereas those who did not receive additional radiotherapy had a median OS of 12 months [63]. A retrospective analysis found a median OS of 19.9 months, compared to 10.9 months in the control group receiving RT with concurrent TMZ [64]. Finally, results from two completed Phase 1 studies (NCT03416530 and NCT03134131) demonstrated that, if patients were treated with ONC201 following the first radiation treatment but before recurrence, median overall survival increased to 21.7 months vs. 9.3 months if treated after recurrence [65]. This suggests that the timing of ONC201 therapy is crucial. Further clinical trials are underway, including a Phase 2 trial of ONC201 combined with panobinostat or paxalisib, a PI3K/mTOR inhibitor (NCT05009992). Additionally, a multicenter, randomized, open-label Phase 3 trial (NCT05476939) is comparing the effect of ONC201 to the mTOR inhibitor everolimus [66].

### 4.3. Immunotherapies

Immunotherapeutic approaches to DMG pose unique challenges. DMG is considered an immunologically “cold” tumor, even when compared to GBM [32]. Also of note, the diffuse location of most DMG tumors in critical midbrain and brainstem structures increases the likelihood of morbidity secondary to immunogenic therapies [67]. Striking a balance between upregulating the anti-tumor immune response and minimizing the risk of inflammation and edema within the tumor will be vital for the success of immunotherapy in DMG.

A study by Mount et al. identified high expression levels of the disialoganglioside GD2 on H3K27M DMG cells as a potent target for chimeric antigen receptor (CAR) T cells; GD2-directed CAR T cells showed promising preclinical efficacy in patient-derived DMG models [68]. A 2022 dose-escalation Phase 1 trial of these GD2-directed CAR T cells showed that, while some patients exhibited “tumor inflammation-associated neurotoxicity”, the therapy showed a moderate OS benefit compared to historical controls [69]. This trial remains ongoing (NCT04196413). Other studies investigating the effect of CAR T-cell-based therapies for DMG target the tumor surface antigen B7 homolog 3 protein (B7-H3); a Phase 2 study of B7-H3 CART cells is underway (NCT04185038) [70]. DMG tumors have also been shown to highly express HER2, and HER2 CAR T cell therapy has shown efficacy both in vitro and in vivo. This has led some to suggest that CAR T-cell clinical trials for pediatric patients with brain tumors should be expanded to include patients with DMG tumors [71]. 

Immune checkpoint inhibition via programmed cell death protein 1 (PD1) inhibition with nivolumab did not show a survival benefit in DMG [72]. Convection-enhanced delivery (CED) of GB-13 (IL13.E13K-PE4E), targeting the interleukin 13 receptor subunit alpha 2 (IL-13 Rα2), showed promising preclinical results in vivo [73]. Convection-enhanced delivery bypasses the BBB via catheters connected to pumps that create a continuous pressure gradient and drive the flow of the drug solution into the tumor [74]. Finally, vaccine-based approaches to DMG are under early investigation; preclinical findings validated H3K27M as a target to induce immune responses to a peptide vaccine against DMG cells in a major histocompatibility complex (MHC)-humanized mouse model [75]. A first-in-human, compassionate use trial of a H3K27M-specific long peptide vaccine in eight adult DMG patients showed that the vaccine was safe and immunogenic in human patients [76]. A multicenter Phase 1 trial for this peptide vaccine is now underway (NCT04808245) [77].

## 5. Improving Intratumoral Drug Delivery in H3 K27-Altered DMG

**Figure 3:**
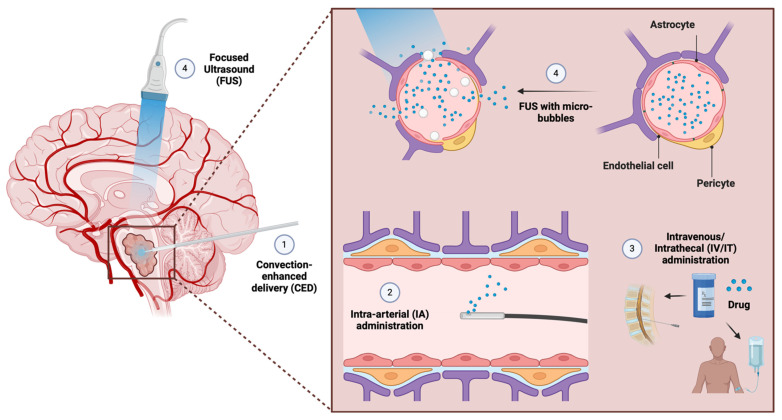
Methods of improving intratumoral drug delivery in DMG. 1. Convection-enhanced delivery (CED) bypasses the blood–brain barrier (BBB) to deliver drugs via micro-catheters and a positive pressure gradient. 2. Intra-arterial administration can directly deliver drugs to a target artery that feeds the tumor. Osmotic agents can be added to disrupt the blood–brain barrier. 3. IV/IT drug administration can overcome at least some first-pass metabolism and, for IT administration, bypass the BBB. 4. Focused ultrasound (FUS) can non-invasively and temporarily disrupt the blood–brain barrier; this technique can be combined with microbubbles for increased efficacy.

The BBB remains a key therapeutic challenge in DMG [33,78]. The BBB is composed of endothelial cells (EC), capillary basement membranes, pericytes, and astrocytic end feet [79]. These structures, as well as transporters and efflux pumps present on endothelial cells, conspire to limit the passage of xenobiotics and other foreign substances into the brain. Paracellular movement of drugs and/or metabolites is regulated by tight junctions between ECs, whereas transcellular passage is regulated by efflux pumps and transporters [80]. The integrity of the BBB and blood–brain tumor barrier (BBTB) is significantly but heterogeneously disrupted in brain tumors such as glioblastoma [81,82]. In contrast, however, studies suggest that the BBB/BBTB is well preserved in DMG [33,83]. This impermeability contributes to the hypointensity of these tumors on contrast-enhanced T1 MRI, potentially making early diagnosis challenging [84]. Furthermore, this preserved BBB integrity makes achieving high doses of effective chemotherapies in DMG extremely challenging. In order to administer targeted therapies at effective intratumoral doses, novel drug delivery strategies, including CED, FUS, and intra-arterial delivery, are being tested in DMG (Figure 3).

### 5.1. Targeted Drug Delivery Systems

Systemic drug delivery typically does not achieve significant intratumoral drug concentrations in DMG. Furthermore, administering high systemic doses of chemotherapeutic agents in an attempt to increase the intratumoral dose often leads to systemic toxicity, particularly in pediatric patients [85]. CED, which bypasses the BBB via direct placement of micro-catheters and the creation of a positive pressure gradient for drug solutions within the tumor, provides a mechanism for direct interstitial drug delivery in DMG. This method produces higher intratumoral drug concentrations and limits the adverse systemic effects of the treatment [86,87,88]. Encouraging preclinical studies found beneficial effects of CED-administered drugs, such as the HDACi panobinostat, the EZH2 inhibitor EPZ-6438, and several PI3K/MEK inhibitors [54,89,90]. In 2018, Souweidane et al. published a dose-escalation study with 28 DMG patients and found CED to be a rational and safe therapeutic approach. As expected, CED achieved high intratumoral drug concentrations in this study [34]. Current ongoing trials (NCT03566199 and NCT04264143) are investigating the efficacy of MTX110, a nanoparticle form of panobinostat administered via CED in DMG [78]. Several variables must synergize in order to achieve successful drug delivery with CED. Besides drug efficacy, infusion parameters, and potential clearance mechanisms from the brain, the volume of distribution of the drug is an important variable that cannot be ignored [91].

Intrathecal (IT) and intraventricular (IV) drug administration are well-established ways to achieve high drug concentrations in the CNS. These methods can overcome at least some first-pass metabolism and, in the case of IT administration, the BBB [92]. The IT application of methotrexate (MTX) is commonly used in CNS metastatic acute lymphatic leukemia and lymphomas [93,94]. Several trials assessing IV or IT as drug delivery strategies for pediatric brain tumors, such as ependymoma and medulloblastoma, are underway (NCT04958486). To date, multiple trials have provided evidence for the feasibility and safety of IV/IT applications of various drugs [95,96]. However, factors limiting efficacy, such as poor parenchymal penetration and rapid brain clearance rates, mitigate the success of IV/IT in the context of pediatric brain tumors [97]. The potential of combining IV/IT administration with nanotechnology in an effort to increase its efficacy is being actively explored [98]. 

In addition to the IV and IT approaches, neuroendovascular methods can be used to reach an artery supplying the tumor (i.e., a target artery) and directly deliver a drug through that vessel. Intra-arterial (IA) administration of drugs can achieve a more targeted administration and thus minimize systemic side effects [99]. The use of osmotic agents such as mannitol in IA administration can increase BBB permeability during IA drug administration. Osmotic agents induce endothelial cell shrinkage and paracellular permeability, thereby increasing delivery to the brain parenchyma [100]. Delivery of chemotherapy with IA or IA combined with osmotic BBB disruption was found to be safe in a study of 436 adult patients with brain tumors [101,102]. Similarly, IA delivery of bevacizumab and cetuximab combined with mannitol to induce BBB opening in DMG was found to be safe in a Phase 1 clinical trial [42]. Of note, this trial used SIACI. This precise method, which increases intratumoral drug concentration and mitigates systemic toxicity by careful target artery selection, is especially appropriate for the delicate anatomical location and complex blood supply of DMG tumors [103]. RMP-7, a bradykinin analog, was also explored as an alternative mechanism for increasing drug uptake into gliomas [104,105]. However, a Phase 2 study of RMP-7 combined with intravenous carboplatin found no evidence for increased efficacy of the combined approach [106]. 

Finally, FUS combined with IV-administered lipid, protein, or polymer microbubbles can non-invasively and temporarily disrupt the BBB. Specifically, the intravascular mechanical forces generated by the microbubbles induce transient openings in tight junctions, thereby increasing BBB permeability [35,107,108]. In 2021, MRI-guided FUS was shown to deliver the monoclonal antibody trastuzumab safely and effectively to adult patients with brain metastases [109]. FUS has been well-tolerated in preclinical in vivo models of DMG [35,110]. FUS has also been used to enhance the intratumoral drug concentrations of etoposide and doxorubicin in these disease models [35,111]. Tumor growth rates in DMG PDX models have been successfully suppressed using FUS, and a recent study using a combination of panobinostat and FUS combined with microbubbles (FUS/MB) significantly increased survival in a DMG mouse model [112,113]. An ongoing Phase 1 clinical trial is investigating the potential benefit of FUS/MB BBB disruption and panobinostat treatment in DMG patients that received radiotherapy (NCT04804709) [78]. A separate Phase 1 study is examining the feasibility of using FUS to open the BBB in pediatric DMG patients receiving oral etoposide (NCT05762419). Finally, an ongoing Phase 2 study of sonodynamic therapy in combination with MRI-guided FUS (NCT05123534) has enrolled 37 patients.

### 5.2. Nanoparticles

Nanoparticles (NP) are small constructs ranging in size from 1 to 100 nanometers. They can be broadly categorized into three subgroups: polymeric, inorganic, and lipid-based [114]. NPs can encapsulate drug cargo, increasing the stability, solubility, and transport potential of their contents [114]. These modular constructs may also be engineered to better penetrate biological barriers, including the BBB, thereby promoting the delivery of drugs to the brain [115]. The FDA has approved several NP formulations for the delivery of chemotherapeutics, with applications in acute myeloid leukemia, pancreatic cancer, metastatic breast cancer, and more [116,117,118,119,120,121]. Of note, NP formulations have faced significant translational challenges in brain cancer, primarily due to difficulty achieving sufficient intratumoral drug concentrations [122]. NP-encapsulated therapies may thus benefit from mechanisms that assist in bypassing the BBB, including CED, FUS, and IA administration.

NP chemotherapy formulations are under active investigation in preclinical models of pediatric brain cancer. Pox-Palbo, a NP formulation of the CDK4/6 inhibitor palbociclib, was shown to improve the drug’s pharmacokinetics in the brain and prolong survival in a mouse model of medulloblastoma driven by the SHH mutation [123]. Fucoidan-based NPs containing vismodegib achieved similar effects [124]. However, these NPs alone were not sufficient to overcome therapeutic resistance in medulloblastoma models, highlighting the need for a combinatorial approach to therapy. 

NP drug formulations have shown some promise in DMG as well. Early work demonstrated that CED of NPs loaded with DNA repair inhibitors could induce radiosensitization in a rat model of DMG [125]. Shargh et al. developed a N(3)-propargyl derivative of TMZ (N3P) for use in DMG models, as DMG tumors are canonically resistant to TMZ therapy. When packaged into either apoferritin nanocages or liposomes and delivered via CED, N3P inhibited DMG growth in vitro [119]. Furthermore, drug-loaded NPs labeled with zirconium-89 showed improved brain distribution/retention in rats compared to free 89Zr. Finally, intranasal delivery of a liposomal formulation of irinotecan, CPT-11, and its active metabolite, SN-38, slowed tumor growth and prolonged survival in a mouse PDX model of DMG [126]. As a result of these promising preclinical data, therapeutic applications of NPs in DMG have recently begun clinical trials. A Phase 1 study investigating a NP-based formulation of panobinostat (MTX110) via CED in DMG patients (NCT04264143) concluded in December 2023, and additional Phase 1 trials of this compound are underway (NCT05324501 and NCT04264143) [78].

## 6. Future Directions

Despite enormous effort, more than 100 clinical trials of therapeutics in DMG have failed to demonstrate meaningful survival benefits [127,128]. Palliative radiation therapy remains the only beneficial intervention. Though many targeted agents show promise in vitro and even in in vivo DMG models, the aggressive nature, intact BBB/BBTB, immunological senescence, and chemotherapeutic resistance of DMG conspire to dash the success of in-human trials. The challenges in translating preclinical success to clinical efficacy are multifaceted. The eloquent anatomic location and diffusely infiltrative growth pattern of DMG present significant hurdles. Furthermore, variations in tumor size, drug penetrance, and drug efflux between preclinical models and human patients, along with toxicity concerns, contribute to the limitations in successfully translating novel DMG therapies to bedside applications. Recognizing the unique obstacles posed by DMG in children compared to preclinical models is crucial for informing future drug development initiatives. Strategies for combating this array of therapeutic challenges may include combination drug therapies, concurrent use of multiple therapy types, and disruption of the BBB as therapy is administered.

While various studies have demonstrated stereotactic biopsies to be safe and a valuable diagnostic tool in selected DMG patients, less invasive and safer methods to diagnose and monitor DMG are needed, especially in the context of disease and treatment monitoring. The analysis of various biomarkers in biofluids, such as blood, CSF, or urine, has gained popularity across different diseases. These so-called liquid biopsies have shown great potential for DMG. Considering the delicate location of DMG and its selective BBB/BBTB, the concentration of DMG biomarkers is higher in CSF compared to patient plasma, making CSF the most useful liquid biopsy in DMG patients, although ongoing work investigates the utility of plasma as a liquid biopsy in DMG. DMG-derived, H3K27M mutant circulating tumor DNA (ctDNA) and variant allele fraction (VAF) in CSF and plasma can be measured and quantified via droplet digital PCR. Studies have demonstrated the utility of H3K27M mutant ctDNA in evaluating tumor growth and response to treatment. Studies have successfully tested the utilization of H3K27M ctDNA VAF for therapy monitoring in DMG patients treated with ONC201. Future investigations will provide more insights into the usage of liquid biopsies in DMG, not only as a diagnostic tool but also as a tool to study DMGs better.

## 7. Conclusions

DMG stands as a prominent cause of brain tumor-related deaths among children, prompting an urgent call for improved outcomes for patients and their families. This universally fatal malignancy is marked by inherent treatment resistance, diffuse invasion into critical CNS structures, and the challenges posed by a relatively intact BBB/BBTB. These characteristics collectively act as formidable barriers to the development of effective therapies, and novel therapeutic strategies should seek to specifically tackle these unique challenges. Current advances in tackling these challenges are embodied in efforts to find ways to improve drug delivery by breaching the BBB/BBTB and exploring novel drug packaging methods. Combining different therapeutic avenues and evaluating targeted therapies are promising strategies and are a key component in a shift towards innovative treatment strategies for DMG. Through the advancements in our understanding of DMG biology and its molecular features, future studies will be able to evaluate more specific and targeted therapies. By embracing innovative therapeutic modalities, overcoming the BBB barrier, and/or exploring new drug packaging methods, there is hope for providing cautious optimism to patients and families grappling with the devastating impact of DMG.

## Data Availability

No new data were created or analyzed in this study. Data sharing is not applicable to this article.
